# Atomic Mineral Characteristics of Indonesian Osteoporosis by High-Resolution Inductively Coupled Plasma Mass Spectrometry

**DOI:** 10.1100/2012/372972

**Published:** 2012-04-30

**Authors:** Zairin Noor, Sutiman Bambang Sumitro, Mohammad Hidayat, Agus Hadian Rahim, Akhmad Sabarudin, Tomonari Umemura

**Affiliations:** ^1^Department of Orthopaedics, Ulin General Hospital, Faculty of Medicine, Lambung Mangkurat University, Banjarmasin 70232, Indonesia; ^2^Department of Biology, Faculty of Science, Brawijaya University, Malang 65145, Indonesia; ^3^Department of Orthopaedics, Syaiful Anwar General Hospital, Faculty of Medicine, Brawijaya University, Malang 65122, Indonesia; ^4^Department of Orthopaedics, Hasan Sadikin Hospital, Faculty of Medicine, Padjadjaran University, Bandung 40161, Indonesia; ^5^Department of Chemistry, Faculty of Science, Brawijaya University, Malang 65145, Indonesia; ^6^Division of Nanomaterial Sciences, EcoTopia Science Institute, Nagoya University, Nagoya 464-8603, Japan

## Abstract

Clinical research indicates that negative calcium balance is associated with low bone mass, rapid bone loss, and high fracture rates. However, some studies revealed that not only calcium is involved in bone strengthening as risk factor of fracture osteoporosis. Thus, in this report, the difference of metallic and nonmetallic elements in osteoporosis and normal bones was studied by high-resolution inductively coupled plasma mass spectrometry (HR-ICP-MS). The influence of these elements on bone metabolic processes is also discussed. Inclusion criteria of bone samples consist of postmenopausal woman, trabecular bone fracture, normal and osteoporosis BMD value, and no history of previous disease. The results showed that the concentration of B, Al, S, V, Co, Mo, Te, Ba, La, Ni, As, and Ca/P ratio is higher in osteoporosis than normal. These atomic minerals have negative role to imbalance between bone resorption and bone formation activity. Conversely, concentrations of Na, Mg, P, K, Ca, Cr, Pd, Ag, Mn, Fe, Cu, Zn, Rb, Sr, Pb, and Se are lower in osteoporosis than in normal bones. Among these atoms, known to have important roles in bone structure, we found involvement of atomic mineral and calcium which are considerable to contribute to osteoporotic phenomena.

## 1. Introduction

Decreasing of skeletal strength and increasing of fragility and fracture risk was a marker of osteoporosis. This condition is caused by abnormalities of bone density and bone microstructure [1, 2]. A few studies focusing on osteoporosis epidemiology in less developed or developing countries make the prevalence unclear [3]. The prevalence of osteoporosis in South East Asia was estimated of about 15.3% [4]. White paper from Indonesian Osteoporosis Association shows that prevalence of osteoporosis at 2007 was 28.85% for man and 32.3% for woman [5]. 

Organic and inorganic components are responsible for toughness and rigidity of bone, respectively. Both of these substances also show the mechanical strength of the bone. Trabecular bone is more sensitive to hormones or other biological factors that are involved in modulating bone metabolism [6]. Atomic mineral is the smallest component of trabecular bone. Calcium is the most prominent mineral in bone which determines the risk of osteoporosis. Clinical research indicates that negative calcium balance often occurs in aged men and postmenopausal women due to several factors. It appears to be associated with low bone mass, rapid bone loss, and high fracture rates [7]. Several researches show inconsistent results. Prentice shows that in Gambia population who have low calcium daily intake were found to have lower osteoporosis prevalence [8]. Western population whose calcium daily intake is of high level were higher in fracture incidence, conversely in Asian population whose low calcium was lower in fracture incidence [3, 9]. This phenomenon indicates that not only calcium is involved in bone quality as a risk factor of fracture osteoporosis.

Each atomic mineral was able to substitute another atomic mineral due its similarity in the atomic radius. Ren et al. showed that Zn substitution on hydroxyl apatite crystal was found to inhibit crystal growth due to its smaller atomic radius as compared with Ca [10]. This finding was also confirmed by Miyaji et al. [11]. Moreover, several publications indicate the importance of atomic composition in bone. Ščančar et al. explore the composition of mineral in *iliac crest* autopsy sample by *atomic absorption spectrometry* (AAS) and conclude that Zn, Rb, Se, Fe, Al, Cu, Pb are in mg/g level, and Ca is in g/mg level [12]. Brodziak-Dopierala et al. measure Ni, Mn, Cr, Ca, Pb, Cu, Fe, Zn, Mg, K, Na, and Cd in several head femur sections which indicate its effect on mechanical stress [13]. Zaichick and Tzaphlidou say that Ca/P ratio is the best predictor to explain bone disorder [14]. Alfvén et al. conclude that low exposure of cadmium increases osteoporosis risk [15]. However, almost all previous papers explored only few atomic minerals and little association with osteoporosis. 

So far, no detail information is dealing with the bone quality of Indonesian people. We just believe that its quality is not solely determined by calcium, but no explanation as an answer of the hypothesis of why in Indonesian population the fracture rate of osteoporosis are low does not in accordance with the fact of low Calcium intake. Accordingly, the objective of this study is to investigate the characteristics of atomic mineral in Indonesian osteoporosis and normal bone by HR-ICP-MS. About 27 elements were successfully analyzed, which include B, Al, S, V, Co, Mo, Te, Ba, La, Ni, As, Na, Mg, P, K, Ca, Cr, Pd, Ag, Mn, Fe, Cu, Zn, Rb, Sr, Pb, and Se. The present study provides more detail information of atomic mineral compositions as well as better understanding for the association of atomic mineral (beside calcium) with osteoporosis.

## 2. Methods

The study was mainly conducted in the Department of Orthopaedics, Ulin General Hospital of Banjarmasin and the Department of Orthopaedics, Syaiful Anwar Hospital of Malang from September 2010 to January 2011. Inclusion criteria consist of postmenopausal woman, trabecular bone fracture, normal and osteoporosis BMD value, age less than 50 years, and no history of previous disease. Bone was obtained in surgery room and then analysed for its atomic mineral compositions using HR-ICP-MS (ELEMENT2, Thermo Fisher Scientific, USA) at the Division of Nanomaterial Sciences, EcoTopia Science Institute, Nagoya University, Japan. The mineral measured consists of 27 elements, such as B, Al, S, V, Co, Mo, Te, Ba, La, Ni, As, Na, Mg, P, K, Ca, Cr, Pd, Ag, Mn, Fe, Cu, Zn, Rb, Sr, Pb, and Se. Prior to ICP-MS measurement, dry weight of bone samples were digested with a microwave-assisted acid digestion system (ETHOS E, Milestone General, Italy). Approximately 100 mg of the sample in a PTFE digestion vessel was weighted, into which 4 mL of HNO_3_ and 1 mL of H_2_O_2_ were added. The temperature program of a microwave irradiation was ramped to 220°C within 15 min and kept at this temperature for 10 min. After cooling the vessels, proper amounts of the internal standard solution contained Rh and Re were added. Then, all solutions in the vessels were transferred into polypropylene bottles and diluted with 0.1 M HNO_3_ (ultrapure grade, Kanto chemical, Japan) up to 50 mL. Minimal samples for normal and osteoporosis groups based on Epi Info version 6 were 32 samples for both groups. Statistical analysis was performed using *t* student-test, and *P*  value < 0.05 was considered statistically significant.

## 3. Results

Fifteen osteoporosis BMD and twenty three normal BMD values were involved in this study. HR-ICP MS analytical results showed different concentration of atomic minerals in osteoporosis and normal postmenopausal women. The concentration of B, Al, S, V, Co, Mo, Te, Ba, La, Ni, As, and Ca/P ratio in osteoporosis bones was found higher than that of normal bone as shown in Table 1. Lower concentrations of Na, Mg, P, K, Ca, Cr, Pd, Ag, Mn, Fe, Cu, Zn, Rb, Sr, Pb, and Se were detected lower in osteoporosis than in normal bones as given in Table 2.

## 4. Discussions

Geometrics pattern of bone atomic minerals for substitution or incorporation is based on the properties of atoms in the periodic tables of Mendeleev. Substitution is the replacement of an atom with other atoms due to the similarity of atomic radius and atomic charge. Incorporation is joining an atom to a molecule and/or a composite that can alter the integrity of a molecule. Molecular cavity can also play considerable role in substitution or incorporation of atoms. All of these phenomenons significantly affect the properties and structure of bone crystal.

The concentration of B, Al, S, V, Co, Mo, Te, Ba, La, Ni, As, and Ca/P ratio in osteoporosis is higher than in normal bone. These atomic minerals have negative role in imbalance between bone resorption and bone formation activity. Conversely, the concentration of Na, Mg, P, K, Ca, Cr, Pd, Ag, Mn, Fe, Cu, Zn, Rb, Sr, Pb, and Se in osteoporosis is lower than in normal. Beside that, concentration of B and As is higher significantly (*P* < 0.05) in osteoporosis bone than in normal bone. Concentration of Pd, Rb, and, Se is higher significantly (*P* < 0.05) in normal bone than osteoporosis bone.

As it is well known, plant is a rich source of boron, but meat and fish are poor sources of boron [16]. Effect of boron on bone was still controversial. Nielsen et al. showed that boron deprivation decreased bone volume fraction and increased trabecular separation. Additionally, boron deprivation decreased trabecular thickness [17]. In another case, increased level of boron can strengthen a bone, but it generates fragility. The increase in chemical resistance of calcified tissue, which changes a resorption activity, could also be attributed to the effect of boron in bone [18]. As shown in Table 1, concentration of boron in osteoporosis bone is significantly higher than in normal bone (*P* < 0.05) and indicates decrease in a bone mineral density. Since boron is substitution mineral for phosphorus, the concentration level of phosphorus in osteoporosis bone would be lower than in normal bone as observed in this study. Simulation using crystal maker software shows that the substitution of phosphorus by boron resulted in decreasing atomic density for both osteoporosis and normal hydroxyapatite bones as shown in Table 3. Due to the decrease in atomic density, we expected that this substitution induces amorphous state in the osteoporosis bone. However, this is not proven since the filled space increased along with the decrease in void space after boron substitution as given in Table 4.

Ščančar et al. reported that concentration of aluminum in bone from autopsy subject was 3–10 mg/kg [12]. Effect of aluminum on bone was also still controversial. In vitro study shows that aluminum induced neo-osteogenesis effect via direct stimulation on osteoblast activity [19]. In another case, it was reported that aluminum inhibits bone formation through reduction of osteoblast activity, osteoid mineralization, and matrix formation [20, 21]. In this study, level of aluminum in osteoporosis is higher than in normal bone. We suppose that aluminum is involved in the imbalance of bone formation and resorption activity, probably via reduction of osteoblast activity, osteoide mineralization, and matrix formation.

Vanadium, nickel, cobalt, and arsenic are toxic metals for osteoblast in vitro. High concentration of these metals induces osteoblast apoptosis [19–22]. In this study, vanadium, nickel, cobalt, and arsenic in osteoporosis bone are higher than in normal bone, which may result in the inhibition of bone formation and subsequently contribute to the imbalance condition between bone formation and resorption activity. As vanadium and arsenic are substitution minerals for phosphorus in hydroxyapatite crystal, further investigation to ascertain the effect of vanadium and arsenic substitution for phosphorus in osteoporosis bone was simulated using Crystal Maker software. Although vanadium and arsenic substitution resulted in increasing atomic density (Table 5), however the filled space of hydroxyapatite decreased (Table 6), indicating an increase in crystal porosity.

The highest concentrations of Barium in the body are found in the bone, and it is primarily deposited in areas of active bone growth [23]. The uptake of Barium into the bone occurred rapidly. For example, one day after rats were exposed with barium chloride aerosols, approximately 78% of Barium was found in the skeleton; by 11 days past the exposure, more than 95% of barium was found in the skeleton [24]. In this study, barium in osteoporosis bone was found higher than in normal bone. This data indicates that barium is involved in imbalance between bone formation and resorption in osteoporosis. Barium is substitution atom for calcium in hydroxyapatite that could be found in the osteoporosis bone. With crystal maker simulation, substitution of barium to calcium increases atomic density (Table 7) and decreases porosity of hydroxyapatite osteoporosis bone as indicated by increases in filled space (Table 8).

Iron has a role in bone formation as an enzymatic cofactor for the synthesis of collagen. Calcium could inhibit the iron formation. Beside that, iron is also toxic for bone cell, which can induce osteoporosis by disturbing bone mineralization [18, 25]. Ščančar et al. reported that concentration of iron in bone from autopsy subject was 100–200 mg/kg [12]. In this study, iron level in normal bone is higher than osteoporosis bone. Iron in normal bone is essential for the synthesis of collagen.

Ščančar et al. also found that concentration of copper in bone from autopsy subject was 100–200 mg/kg [12]. Copper makes a crosslink with collagen. Copper was antagonist for zinc in bone, which means that increasing copper induces decreasing zinc level [26]. Copper deficiency decreases osteoblast activity and induces osteoporosis [27]. In this study, we found deficiency of copper in osteoporosis bone. Additionally, there is no inverse correlation between copper and zinc, indicating no toxic effect of these metals on bone cell.

Magnesium was not a main component in hydoxyapatite crystal but was able to join with this crystal. Magnesium is substitution mineral for calcium in composite hydroxyapatite crystal [28]. Magnesium deficiency was a risk factor of osteoporosis [2, 29, 30]. A previous study concluded that magnesium level among osteoporosis subjects was significantly lower than nonosteoporosis [31]. Magnesium acts as a mitogenic factor for bone cell, and its depletion can cause cellular growth inhibition in vitro. Magnesium deficiency induces decreasing collagen formation, sulfation of glycosaminoglycan, and tetracycline labeling [32–34]. In this study, deficiency of magnesium is found in osteoporosis bone. This deficiency probably induces inhibition of cellular growth and decreasing collagen formation in osteoporosis bone. Moreover, it was found that magnesium is not substitution mineral for calcium.

Manganese was prominent atomic for mucopolysaccharide metabolism that affects the organic matrix formation. Manganese was able to substitute calcium due to similar oxidation state. In osteoporosis patient, manganese was found in low level [18, 24]. Prolonged manganese deficiency has been reported to produce skeletal abnormalities, such as osteoporosis [35]. In this study, we found that manganese level in osteoporosis is lower than in normal bone. Low level of manganese is not followed by higher level of calcium in osteoporosis, which indicates that no substitution occurred to these metals in the hydroxyapatite crystal. Therefore, we suppose that manganese functions in the formation of organic matrix.

Zinc has been demonstrated to be an essential element for normal growth of skeleton and bone metabolism. The concentration of zinc in bone is higher than in the other tissues [36]. Zinc is abundant atomic mineral in bone as a prominent element for stimulus of bone formation in vitro and in vivo as well as the inhibition of bone resorption. Zn also supports metabolism and growth of bone, increases bone density, and inhibits bone loss [37, 38]. Zinc, also involved in bonding with organic structure [26]. Our study shows that the deficiency of zinc was found in the osteoporosis bone. This is the reason of imbalance between bone formation and resorption activity in osteoporosis.

Ščančar et al. found that concentration of strontium in bone from autopsy subject was 13–60 mg/kg [12]. According to in vitro study, strontium induces maturation of human primary osteoblast and apoptosis of human primary osteoclast at the same time [38]. Administration of low-dose strontium in rat induces bone formation and in higher-dose strontium would induce mineralization defect [39]. In this study, strontium level in normal bone is higher than in the osteoporosis bone, which is involved in bone formation. In hydroxyapatite crystal, strontium was antagonist for calcium [28]. In osteoporosis and normal bone, there is no converse level between strontium and calcium. It means that there is no substitution of strontium in the hydoxyapatite crystal structure.

Lead in organic or inorganic forms is absorbed through the lungs and gastrointestinal absorption. Organic leads also absorbed through a skin. Inhalation exposure was more common in occupational setting, whereas in the general population through the ingestion route [40, 41]. Up to 94% of the body burden of lead is in bone, where it has a half-life of years to decades [40]. In vitro study shows that in osteoblast, lead would inhibit osteonectin/SPARC calcium binding protein [42]. Lead induces osteoporosis through inhibition of vitamin D and inhibits calcium absorption and disturbing cellular function [43]. In this study, lead level in normal bone is higher than in osteoporosis bone. It is caused by half-life state in decade. Age of osteoporosis subject in this study is twice that of normal subject. In osteoporosis bone, unbounding of lead would disturb cellular function yield imbalance bone resorption and bone formation.

Selenium was a mineral found in soil and organism. Selenium was incorporated into protein as selenocysteine. Selenoprotein was important for bone metabolism. The population with selenium deficiency was found higher in osteoporosis incidence [44]. In this study, we found that selenium level in normal bone is higher than that of osteoporosis bone. It means that selenium is important for bone metabolism and selenium deficiency, which induces imbalance between osteoblast and osteoclast activity. This reason is supported by Moreno-Reyes et al. that the number of osteoblasts and osteoclasts was higher in the selenium-deficient rats and associated with low plasma osteocalcin, a marker of osteoblast activity, and low urinary deoxypyridinoline concentration, a marker of bone resorption. Osteoblast activity was impaired in the selenium-deficient rats, and the increased number of osteoblasts may represent a compensatory process to offset decreased cell activity. The lower plasma osteocalcin associated with low urinary deoxypyridinoline concentrations may indicate that bone formation was depressed in the selenium-deficient rats [45].

Palladium, silver, and lantanadium are atomic minerals for unknown function that need further investigation.

In summary, among these atoms, known to have important roles in bone structure, we found involvement of atomic mineral and calcium which are considerable to contribute to osteoporotic phenomena. These mineral atomics would determine bone mass density. To our knowledge, this study is the first time to provide such evidence. Consideration of involvement of mineral atomic beside calcium in osteoporosis is warranted to prevent osteoporosis.

## Figures and Tables

**Table 1:** Average of atomic mineral concentrations which is higher in osteoporosis bone.

Element	Concentration *μ*g g^−1^		*P* value
	Normal bone	Osteoporosis bone	
B	2.49	4.40	0.04
Al	32.91	290.90	0.48
S	2760.17	2775.24	0.42
V	0.10	0.19	0.12
Co	0.02	0.03	0.07
Mo	0.00	0.01	0.29
Te	0.00	0.01	0.68
Ba	2.61	1138.61	0.86
La	0.00	0.02	0.15
Ni	36.57	43.74	0.10
As	0.00	0.01	0.03
Ca/P ratio	0.95	1.08	0.60

B: boron; Al: aluminum; S: sulphur; V: vanadium; Co: cobalt; Mo: molybdenum; Te: tellurium; Ba: barium; La: lanthanum; Ni: nickel; As: arsen; Ca/P: calcium/phosphorus; *μ*g g^−1^: microgram/gram.

**Table 2:** Average of atomic mineral concentrations which is lower in osteoporosis bone.

Element	Concentration *μ*g g^−1^		*P* value
	Normal bone	Osteoporosis bone	
Na	1248.79	972.75	0.58
Mg	921.06	816.17	0.56
P	63827.16	40757.15	0.42
K	2.02	1.50	0.30
Ca	88964.72	58150.53	0.70
Cr	4.87	1.28	0.82
Pd	10.15	2.40	0.01
Ag	0.08	0.01	0.13
Mn	0.31	0.08	0.70
Fe	224.15	187.32	0.58
Cu	9.39	3.75	0.91
Zn	114.50	47.00	0.60
Rb	1.04	0.32	0.03
Sr	44.90	32.41	0.94
Pb	0.29	0.07	0.58
Se	5.59	1.28	0.02

Na: sodium; Mg: magnesium; P: phosphorus; K: potassium; Ca: calcium; Cr: chromium; Pd: palladium; Ag: argentum; Mn: manganese; Fe: iron; Cu: cuprum; Zn: zinc; Rb: rubidium; Sr: strontium; Pb: plumbum; Se: selenium; *μ*g g^−1^: microgram/gram.

**Table 3:** Effect of boron on atomic density in hydroxyapatite of osteoporosis and normal bone.

	Atomic density/kg m^−3^	
	Normal bone	Osteoporosis bone
Before boron substitution	3213.49	3188.48
After boron substitution	2829.59	2804.58

**Table 4:** Porosity of osteoporosis bone for boron substitution.

	Filled space/%	Void space/%
Before boron substitution	20.71	79.29
After boron substitution	20.84	79.16

**Table 5:** Atomic density in hydroxyapatite of osteoporosis and normal bone.

	Atomic density/kg m^−3^	
	Normal bone	Osteoporosis bone
Before vanadium substitution	3213.49	3188.48
After vanadium substitution	3597.79	3568.76
Before arsen substitution	3213.49	3188.48
After arsen substitution	4050.41	4025.41

**Table 6:** Porosity of osteoporosis bone for vanadium and Arsen substitution.

	Filled space/%	Void space/%
Before vanadium substitution	20.71	79.29
After vanadium substitution	18.40	81.60
Before arsen substitution	20.71	79.29
After arsen substitution	17.92	82.08

**Table 7:** Atomic density in hydroxyapatite of osteoporosis and normal bone.

	Atomic density/kg m^−3^	
	Normal bone	Osteoporosis bone
Before barium substitution	3213.49	3188.48
After barium substitution	5681.91	6274.01

**Table 8:** Porosity osteoporosis bone for barium substitution.

	Filled space/%	Void space/%
Before barium substitution	20.71	79.29
After barium substitution	27.84	72.16
